# Intracapsular Femoral Neck Fractures—A Surgical Management Algorithm

**DOI:** 10.3390/medicina57080791

**Published:** 2021-07-31

**Authors:** James W. A. Fletcher, Christoph Sommer, Henrik Eckardt, Matthias Knobe, Boyko Gueorguiev, Karl Stoffel

**Affiliations:** 1 AO Research Institute Davos, 7270 Davos, Switzerland; Boyko.Gueorguiev@aofoundation.org; 2 Department for Health, University of Bath, Bath BA2 7AY, UK; 3 Cantonal Hospital Graubünden, 7000 Chur, Switzerland; Christoph.Sommer@ksgr.ch; 4 University Hospital Basel, 4052 Basel, Switzerland; henrik.eckardt@usb.ch (H.E.); karl.stoffel@usb.ch (K.S.); 5 Department of Orthopaedics and Trauma Surgery, Lucerne Cantonal Hospital, 6000 Lucerne, Switzerland; matthias.knobe@luks.ch

**Keywords:** algorithm, arthroplasty, femoral neck fractures, hip fractures, fixation, screws, surgical management

## Abstract

*Background and Objectives*: Femoral neck fractures are common and constitute one of the largest healthcare burdens of the modern age. Fractures within the joint capsule (intracapsular) provide a specific surgical challenge due to the difficulty in predicting rates of bony union and whether the blood supply to the femoral head has been disrupted in a way that would lead to avascular necrosis. Most femoral neck fractures are treated surgically, aiming to maintain mobility, whilst reducing pain and complications associated with prolonged bedrest. *Materials and Methods*: We performed a narrative review of intracapsular hip fracture management, highlighting the latest advancements in fixation techniques, generating an evidence-based algorithm for their management. *Results*: Multiple different fracture configurations are encountered within the category of intracapsular hip fractures, with each pattern having different optimal surgical strategies. Additionally, these injuries typically occur in patients where further procedures due to operative complications are associated with a considerable increase in mortality, highlighting the need for choosing the correct index operation. *Conclusions*: Factors such as pathological causes for the fracture, pre-existing symptomatic osteoarthritis, patient’s physiological age and fracture displacement all need to be considered when choosing optimal management.

## 1. Introduction

Femoral neck fractures are one of the biggest diseases of modern times, with lifetime incidences of approximately 1 in 4 for women and 1 in 10 for men [1]. Their prevalence and severity result in vast health and financial burdens; they are the most expensive fractures to treat [2]. Complications remain common given the spectrum of different fracture patterns and the baseline functions of the population affected, and if reoperations are required, mortality rates and financial costs rise considerably [3].

Most hip fractures occur within the capsule of the joint. These fractures pose specific difficulties because of the risk of disruption to the vascularity of the femoral head which can lead to avascular necrosis (AVN). Outcomes, compared to extracapsular fractures, are thus potentially more dependent on the choice and quality of the operation performed. Focusing on intracapsular fracture types, we aimed to create an evidence-based algorithm for the management of an intracapsular hip fracture. We present a narrative review, highlighting the latest advancements in fixation techniques, with an evidence-based algorithm to help surgeons of all grades ensure the best outcomes possible for patients.

## 2. Materials and Methods

Articles related to intracapsular hip fracture management were obtained by the investigators from PubMed, MEDLINE, EMBASE, and Cochrane Central Register of Controlled Trials database searches dating from inception to April 2020—using the following core search terms: Hip fracture management; Hip fracture algorithm; Femoral Neck Fracture management; Femoral Neck Fracture algorithm—with inspection of references of these for further studies following the first round of searches. Inclusion criteria were: all types of articles, those related only to intracapsular fractures, those describing surgical and/or non-surgical management, and those presenting a management pathway or algorithm. Articles were excluded if the full text was not available, if they were not in English or were grey literature.

## 3. Results

**Figure 1:**
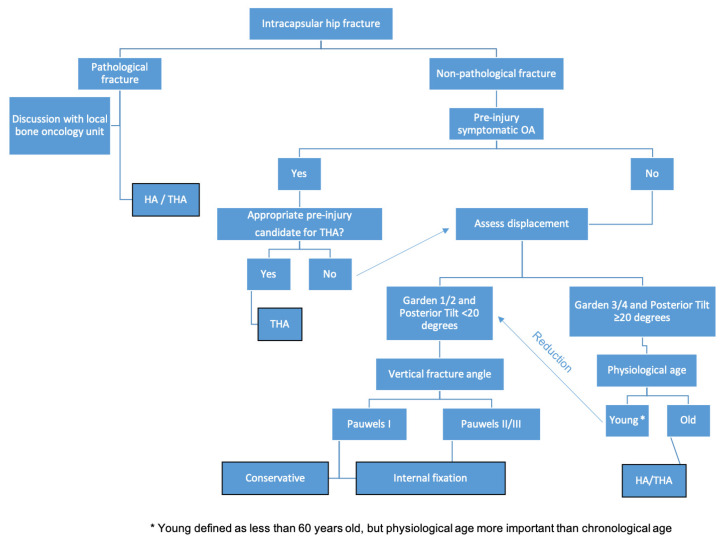
Surgical algorithm for intracapsular femoral neck fractures.

An algorithm generated from the narrative review for managing femoral neck fractures is shown in Figure 1. All treatments should be multidisciplinary, including early orthogeriatric consultation, dedicated femoral neck fracture operative theaters and ensuring adequate pain relief, venous thromboprophylaxis and rehabilitation protocols [4]. Almost all femoral neck fractures are treated surgically [4], with treatment aims remaining constant to enable full unrestricted weight bearing as soon as possible, preserve joint surfaces when feasible, and use implants and prostheses that have a proven design.

## 4. Discussion

Fractures within the region of the femoral neck (AO/OTA 31–B1-3) present surgical challenges due to the uncommon vascular supply to that region. The majority of the blood supply to the femoral head enters in a retrograde fashion, predominantly via the lateral epiphyseal artery, a branch of the medial femoral circumflex artery. Healing of intracapsular fractures occurs through primary osteonal reconstruction due to the inability to bone in this region to form external callus. These anatomical factors combine to heighten the risk of nonunion and AVN with these fractures, further worsened by any disruption to the arterial blood supply due to the energy from the initial trauma, and/or ongoing hypoperfusion through malreduction and reduced arterial flow. However, even in the presence of initial arterial damage, fragment stabilization can be sufficient to reduce the risk of AVN, as reduction and stability enable revascularization across the fracture, before AVN and subsequent collapse can occur [5,6]. Concerns about AVN occurring (including the difficulty in predicting which fractures are more susceptible), fractures not uniting, and the risks from any further operations often justify a more pre-emptive approach in patients with a reduced physiological capacity to deal with such events. This is contrasted with a more accepting stance towards future surgery in young patients [7], where preservation of the native joint predominates due to both the finite lifespan from articular replacements and the increased demands that an active patient would be expected to have.

### 4.1. Presence of Pathological Fracture

Pathological fractures require additional management considerations, in part, due to the lack of healing potential that is required for successful osteosynthesis, alongside presenting other technical complexities such as having reduced quantity and unknown quality of residual bone stock. Therefore, arthroplastic solutions—total hip arthroplasty (THA) or hemiarthroplasty (HA)—are required to restore the patients to their baseline function and to enable mobilization; these operative options have been shown to last longer than fixation treatments [8]. Furthermore, intramedullary procedures may risk generating metastases through embolization of malignant tissue. As further bone destruction can occur after fracture treatment due to the ongoing neoplastic condition, replacement surgery reduces the risk of future reoperation, as may be the case with fixation options. The exception to this is when additional neoplastic lesions within the shaft of the femur would result in stress raisers to an implanted stem. In this instance, cephalomedullary nailing or arthroplasty with concurrent stress reducing plating are surgical options but require planning on a case-by-case basis. Thorough imaging of adjacent joints is required to assess for other lesions and to ensure that stress raisers are neither encountered nor created intra- or post-operatively.

### 4.2. Non-Pathological Fractures—Fracture Displacement

In the absence of prior symptomatic osteoarthritis (OA), fracture displacement needs to be assessed to determine the operative intervention. Completely undisplaced fractures occur rarely [9]. Though being an imperfect surrogate marker [10], displacement is thought to correlate with suspected femoral head blood supply disruption, which in turn predicts the rates of AVN; reportedly between 7 and 78%, with younger groups having higher rates, probably reflecting the higher energy that caused their fracture [9,11,12]. Displacement needs to be assessed orthogonally. Commonly the Garden classification [13] (I—valgus impacted, II—undisplaced, III—partially displaced, IV—fully displaced) is used for assessing coronal displacement, though the key distinction needed is whether there is true displacement or not, regardless of whether partial or full. Firstly, as there has not been shown to be a difference in healing rates between Garden III and IV [14] and secondly, as partially and fully displaced fractures are managed in the same way. Additionally, as the Garden classification is based only on coronal radiographs, lateral images must also be viewed to ensure displacement is not underestimated or missed entirely. Sagittal displacement, typically measured on a lateral radiograph, ≥20° posterior tilt, or ≥10° anterior tilt, has been shown to worsen outcomes when fixed [15,16].

#### 4.2.1. Undisplaced Fractures

Garden I and II fracture patterns and those with <20° posterior tilt/<10° anterior tilt can be categorized as undisplaced given how they can be surgically managed. With these, it is anticipated that the blood supply to the femoral head is either intact and/or there is a low risk of developing AVN, thus in situ fixation enables maintenance of this position against baseline transverse shear and rotational forces and during locomotion [5,6]. To reduce the risk of metalwork failure, estimation of the resultant force acting on the hip due to the fracture line is needed, including whether this is predominately a compressive or a shear force as this will affect the risk of failure from different surgical procedures [17]. Pauwels classification categorizes this, as it is based on the vertical orientation of the fracture line, with type I being less than 30° from the horizontal, II—30–50°, and III—more than 50° [18]. Impacted fractures can be managed conservatively, with reduced weight bearing and close follow-up for signs of displacement. However, this strategy can be associated with an increased risk of nonunion, subsequent surgery and secondary instability, thus prophylactic fixation is recommended and usually performed. Surgically fixed, undisplaced fractures have an extremely high rate of union (>95%), with low rates of AVN (<5%) and need for any future operation [19], especially when compared to cases managed non-operatively [20].

##### Fixation Methods for Undisplaced Fractures

The aim of fixation is to allow compression at the fracture site without shortening, whilst maintaining alignment. Fixation options have typically been either multiple cancellous cannulated screws or a sliding hip screw (SHS). The former offers benefits of a smaller biological footprint both from the surgical exposure and implanted metalwork, whilst the later provides greater angular stability [21]. There is limited clinical evidence of an advantage of one fixation method over another [22]. A previous review found no superiority between cannulated screws osteosynthesis and SHS, though an increase in AVN with cannulated screws [23]. However, a more recent prospective trial randomizing 1079 unblinded patients to receive either SHS or cancellous screws contradicts the increase in AVN, though agrees that overall, there is no superiority between either method [24]; it showed a higher rate of AVN and more conversions to THA after SHS fixation, though no difference in patient reported outcomes. SHS led to better outcomes in current smokers, more displaced fractures, and fractures with a more vertical line (Pauwels III), explained by the angular stability created by this construct.

With cannulated screws, typically two to four are placed for fixation of femoral neck fractures. Restoration and engagement of the medial ‘calcar’ arch is needed for best outcomes. Three screws have been shown to be biomechanically better than two [25,26], however, no clinical difference has been demonstrated purely based on the number of used screws [23]. In fact, the crucial factor in achieving bone union appears to be the location of screw placement rather than the number of screws [27]—although more screws may increase the chances of correct placement. If two screws are inserted and both are located within 3 mm distance from the cortical bone that forms the inferior arch of the femoral neck, the rate of reported nonunion is 11%, if one is not, this increases to 41%, and if neither of the screws are, the rate is 100% [28]. Additionally, constructs where the screws are oriented more vertically provide greater stability due to increased lateral femoral cortical bone engagement and reduced moment arm forces [29]. A recently developed technique highlights the benefits achievable with optimal screw placement. The biplane double-supported screw fixation (BDSF) method [30] involves inserting three screws on two vertical oblique planes that medially diverge in the direction of the femoral head in lateral view. The lateral insertion points of the screws are more distal compared to traditional cancellous screws, enabling engagement of thicker lateral femoral cortices. For two of the screws—the middle and the distal—these entry points act as the first of two supporting points, with the second being the distal femoral neck cortex. For the distal screw, there is also a third supporting point from the posterior femoral neck cortex. Acting as beams within the neck and head, the former two screws provide resistance to vertical loads, the distal screw counteracts anteroposterior bending forces, and resistance to tensile forces is achieved through the third (proximal) screw. This technique has shown considerable biomechanical advantages over standard cancellous screw placement [29], and when used for displaced fractures in 207 patients was clinically associated with very low complication rates, such as an AVN rate of only 0.5% [31]. New implants have been developed primarily for extracapsular hip fractures, such as the X-Bolt (X-Bolt, Dublin, Ireland) using deployable flanges, and show biomechanical improvements over SHS, especially for rotational stability in low density bone [32]. However, clinical validations of these findings are awaited in intracapsular fractures. A recent approach is the combination of a blade and a screw in one single implant, which is placed on the market as “rotationally stable screw-anchor (RoSA)” [33,34]. In a first clinical setting, the fixation of unstable trochanteric femur fractures using the RoSA in combination with a trochanteric stabilizing plate (TSP) led to a great primary stability, with significant advantages with regard to limited femoral neck shortening [35]. However, a clinical evaluation of this innovative implant in femoral neck fractures is still missing too.

Regardless of the fixation technique used, fixation failure is seen in 5–30% of cases, often requiring salvage procedures [11,36,37,38,39], typically, conversion to hip arthroplasty. Pre-existing OA has been associated with increased fixation failure rates; perhaps as stiffer, arthritic joints place greater stresses on peri-articular implants [38]. Salvage procedures lead to worse outcomes than those of patients who had THA as their index procedure at the time of the fracture [40]. With increasing longevity of primary THA, an increasing role for THA instead of fixation may be seen if the risk of AVN is especially high. Equally, avoiding the risks of fixation failure altogether might be more appropriate when viewing the topic pragmatically in all surgeons’ hands, given the additional operative difficulties fixation can present [41].

#### 4.2.2. Displaced Fractures

##### Patient’s Physiological Age

Displaced fractures—Garden III and IV [13] and/or those with sagittal fracture tilting [15]—occur in approximately 32% of low energy femoral neck fractures [24]. In chronologically young patients, a bimodal mechanism of injury is seen, with one group being predominately, premorbidly well patients involved in high energy trauma and the other group being patients with chronic diseases [42] that have affected their bone density, despite being chronologically young suffering low energy injuries. Assessment of a patient’s comorbidities determines how assertively joint preservation should be pursued. Whilst 60 years of age has been suggested as a chronological cut off between young and old [7], detailed assessment of patient factors is needed to balance the risks and benefits for fixation compared to replacement. In the physiologically younger patient, reduction should be attempted, sufficient to convert a displaced fracture into an undisplaced variant, and then managing the injury as per the undisplaced algorithm pathway. An overarching consideration is whether reduced weight bearing is needed in the initial stages of fracture fixation, to prevent unnecessary loading of the fixation. If compliance with such post-operative instructions would be poor, for example due to neuromuscular conditions or learning difficulties, fixation failure rates may increase, thus negating the benefits of joint preservation. In these instances, primary replacement should be considered despite young chronological age.

##### Young Patients

Patients under 60 years of age account for between 4 and 13% of intracapsular hip fractures [43,44]. However, this group represents the greatest operative challenge as preservation of the native hip joint should be prioritized—due to the demands that may be placed on the implants and as the patient’s life expectancy is greater than that of the implants. The strategy should be to reduce the fracture, converting the configuration into the equivalent of an undisplaced variant and then managed as per this fracture type. Albeit observing low energy fractures, a clinical study on 1059 patients observed that 58% of cases (614 patients) needed some reduction; 5% (48 patients) received open and 53% (564 patients) required closed reduction [24]. For closed reduction, traction in extension with internal rotation of the leg from an abducted position can adequately reduce the fracture [45]. Open reduction may be needed if there is more displacement, as seen after high energy trauma. For open reduction, either an anterolateral or modified anterior approach, or combination can be used, where reduction is performed anteriorly and fixation laterally. No comparisons between the different reduction techniques have been performed; the key being that reduction is achieved rather than the method leading to it.

##### Old Patients

The vast majority of old patients have replacement surgery if they have sustained a displaced intracapsular fracture—92% in the UK [4]. Compared to HA for displaced fractures, osteosynthesis has been shown to result in higher complication rates of between 10 and 45% [20,46,47], including nearly a threefold increase in revision surgery (4% after HA and 11% after fixation of displaced fractures [48]), though with newer fixation techniques these rates have been considerably reduced. Functional outcomes are also better after HA compared to osteosynthesis in the elderly population [49].

Replacement options are either HA or THA. The latter is suggested for patients who were able to walk independently out of doors with no more than the use of stick, are not cognitively impaired and are medically fit for the anesthesia and the procedure; in the UK, 7% of all femoral neck fracture patients received a THA in 2019 [4]. Additionally, THA is indicated in symptomatic pre-injury OA, as a failure to treat prior OA that was limiting the patient’s mobility before the injury can be expected to impact their rehabilitation potential following hip fracture surgery. As the vast majority of hip fractures occur in the elderly, pre-existing OA of this joint is frequently encountered. However, the key information regarding management options is how symptomatic this is during their activities of daily living. Unfortunately, data are lacking and conflicting regarding the medium- and long-term comparisons of HA and THA. No difference in reoperation rate, function or mortality at 2 years has been seen in patients randomised between HA or THA [50], nor in functional outcomes at 12 years [51], however, a meta-analysis has shown improved patient reported outcomes with THA [52], with another review challenging this [53]. Outcomes following THA for displaced fractures have been shown to be worse than following elective THA [40] and are associated with higher mortality rates when performed after trauma rather than electively [54].

Non-operative management in displaced fractures has been considered in high anesthetic risk, low demand patients, with mortality rates at one year being shown to be similar to those who are operated [55]. In a small study of conservatively managed patients, 6 out of 22 patients were able to mobilize without walking aids at one year [55]. However, as the mortality rate was higher with non-operated patients in the initial period and the same at one year, it can be viewed that surgery should be considered in all but patients who will die imminently—as surgery will greatly increase the potential for mobility with a reduction in mortality and pain. Even in the group at highest anesthetic risk, the pain relief achieved from operating, both for the patient at rest and during nursing care, could justify the risks of perioperative death. However, there are clear tendencies to a better overall result in patients receiving multidisciplinary orthogeriatric treatment, with lower rates of cardiorespiratory complications and mortality [56,57]. With an adjusted 30-day mortality being 22% lower for patients with orthogeriatric care, co-management should be encouraged in the treatment of hip fractures [51].

## 5. Conclusions

Intracapsular hip fractures are heterogeneous both in terms of fracture characteristics and patients they occur in. Using the described algorithm enables an evidence-based approach, addressing the numerous factors that need consideration for optimum management.
